# Supplementary material for: Variability of a job search indicator induced by operationalization decisions when using digital traces from a meter
Source: PLoS One. 2025 Dec 22;20(12):e0338894. doi: 10.1371/journal.pone.0338894 (PMC12721509; doi:10.1371/journal.pone.0338894)
Supplement: Attachment — Submitted filename: Rebuttal letter v4.docx [file pone.0338894.s002.docx]

Dear Dr. Ochoa Gómez,

Thank you for submitting your manuscript to PLOS ONE. After careful consideration, we feel that it has merit but does not fully meet PLOS ONE’s publication criteria as it currently stands. Therefore, we invite you to submit a revised version of the manuscript that addresses the points raised during the review process.

Based on the comment of the reviewers, kindly go thoroughly and check their comments on different parts of the manuscript that needs attentive attention. You will find the comments on this email.

We look forward to receiving your revised manuscript.

Dear Editor,

Thank you for your consideration and for the opportunity to revise our manuscript. We are grateful to the reviewers for their thoughtful and constructive comments, which we believe have helped us improve the quality and clarity of the paper.

We have carefully considered all comments and addressed them in detail in our revised version. As multiple comments touched on similar points, we have, in some cases, addressed them through the same set of revisions.

Before presenting our detailed responses, we would like to briefly clarify our position regarding the main concern raised by Reviewer #2, who questioned the contribution and analytical depth of the study.

We acknowledge that our research does not aim to offer a definitive solution to the problem of operationalizing behavioral concepts using digital trace data. Rather, the central motivation of this paper is to draw attention to a fundamental methodological gap emerging in the current landscape of digital behavioral research. Specifically, while different types of digital traces (e.g., metered browsing data, geolocation data) are increasingly used for studying human behavior, they are often treated as de facto gold standards (e.g., Kobayashi & Boase, 2012; Junco, 2013; Araujo et al., 2017) and analyzed without the rigorous methodological scrutiny traditionally applied to survey-based research, particularly in terms of:

1. Unacknowledged sources of error – such as the undercoverage of observed devices, device sharing, or incomplete capture of online activity (especially within-app behavior). These sources can introduce systematic bias but are so far rarely documented or quantified.
2. The lack of a structured operationalization framework – unlike survey research, which follows established procedures for defining and measuring constructs (e.g., concept definition, distinction between concepts by intuition and concepts by postulation, scale selection, and wording refinement), digital trace research lacks comparable methodological guidance.

Our study therefore aims to illustrate the consequences of this methodological gap by systematically demonstrating how seemingly minor operationalization choices can lead to substantial variability in research outcomes.

We fully agree that the next step for the field is to (1) measure the magnitude of these errors and (2) establish strategies to achieve the most effective operationalizations (i.e., those that introduce the lowest levels of error). These steps would constitute practical solutions for researchers seeking first to control and then to reduce such errors. However, we believe that before these solutions can be effectively developed, it is essential to document and quantify the scope of the problem. In this sense, our contribution provides the empirical foundation upon which future methodological standardization can be built.

The manuscript has been revised to make this contribution and its broader implications more explicit throughout the introduction, discussion, and conclusion sections. Although we are not yet able to offer a definitive solution to the problem addressed in this paper, we have sought to provide practical recommendations for researchers who need to operationalize concepts using metered data, in line with the reviewers’ suggestions.

Thank you again for your consideration.

Sincerely,

The Authors

References:

Araujo, T., Wonneberger, A., Neijens, P., & De Vreese, C. (2017). How Much Time Do You Spend Online? Understanding and Improving the Accuracy of Self-Reported Measures of Internet Use. Communication Methods and Measures, 11(3), 173–190. https://doi.org/10.1080/19312458.2017.1317337

Junco, R. (2013). Comparing actual and self-reported measures of Facebook use. Computers in Human Behavior, 29(3), 626–631. https://doi.org/10.1016/j.chb.2012.11.007

Kobayashi, T., & Boase, J. (2012). No Such Effect? The Implications of Measurement Error in Self-Report Measures of Mobile Communication Use. Communication Methods and Measures, 6(2), 126–143. https://doi.org/10.1080/19312458.2012.679243

Reviewer #1: Below we describe, point by point, the aspects we believe to be most relevant to highlight in the following investigation:

Thank you for your feedback on both the strengths and weaknesses of our research. In the responses below, we focus specifically on the suggestions for improvement and the measures we have taken to strengthen our paper accordingly.

Introduction
Strengths:
• The introduction effectively contextualizes the study within the broader challenges of measuring online behavior and the limitations of survey-based approaches.
• The rationale for using metered data is well-articulated.

Suggestions for Improvement:
• The concept of “job search intensity” is introduced but not sufficiently grounded in theory. A more nuanced discussion of its dimensions (e.g., effort, persistence, engagement) would strengthen the conceptual foundation.
• Consider explicitly linking the concept to the metrics used later in the study to improve coherence between theory and operationalization.

We have revised the final part of the section “1. Introduction” to include a formal definition of the concept of job search intensity and to highlight the basic dimension considered fundamental to this concept (i.e., time). In addition, we now anticipate the idea—further developed in the next section—that other dimensions may also be involved, particularly when job search activities are conducted online.

“Building on Ochoa and Revilla’s [21] work and using the same dataset, this paper explores the different possible choices that could have been made to operationalize one of these concepts — job search intensity— and whether these choices affect the results. Job search intensity has long been conceptualized as the degree of effort and persistence individuals devote to finding employment. In classical labor economics, it reflects how much time/energy job seekers invest in search activities, influencing the likelihood and speed of re-employment [22, 23]. Later research expanded this view, treating job search intensity as a multidimensional construct that encompasses various factors beyond time spent searching [24]. As job search activities increasingly take place online, the number of potentially relevant dimensions—observable through digital traces, such as the number of job offers viewed or the number of online applications submitted—has also grown. Understanding how these dimensions manifest in digital contexts has therefore become particularly relevant today.”

Background and Literature Review
Strengths:
• The literature review is comprehensive and includes relevant studies on both survey-based and digital trace-based approaches to measuring job search behavior.
• The discussion of prior work on operationalization variability (e.g., Bosch, Faberman & Kudlyak) is insightful and sets the stage for the current study.
Suggestions for Improvement:
• A comparative table summarizing how job search intensity has been operationalized in previous studies could enhance clarity.
• The review could benefit from more discussion on validation strategies used in past research, especially regarding predictive or concurrent validity.

We have revised the Background section. To provide a clearer overview, we added Table 1, summarizing different operationalizations of job search intensity across survey and digital trace data.

**Table 1**

*Different operationalizations of job search intensity in the literature*

| **Authors** | **Data used** | **Operationalization** |
| --- | --- | --- |
| Ellis et al. [24] | Paper survey | Multidimensional construct including time and effort spent, and number of employers contacted. |
| Blau et al. [25] | Paper survey (postal) | Four items assessing general effort (e.g., “Devoted much effort to looking for a job.”), rated on a 5-point Likert scale (“Strongly Disagree” to “Strongly Agree”). |
| Barber et al. [26] | Paper survey (in person and postal) | Two different operationalizations were used:   1. Total number of job search sources used (0 to 8). 2. An operationalization based on two questions:  - Time spent, measured either (1) through an open question asking for hours per week, or (2) via a scale ranging from “one hour or less” to “more than twenty hours.” - Subjective effort, rated on a 5-point scale from “very little effort” to “a great deal of effort.” |
| Saks and Ashforth [27] | Paper survey (postal) | Four items assessing general effort (e.g., “Spent a lot of time looking for job opportunities”), rated on a 5-point Likert scale (“Strongly Disagree” to “Strongly Agree”). |
| Wanberg et al. [28]  Zacher [29] | Paper survey (in person and postal) | Seven items, including "used the Worldwide Web or other computer services to locate job openings". Response options ranged from 1 (“never or zero times”) to 5 (“very often, at least ten times”). |
| da Motta Veiga [30] | Online survey | Four items assessing the extent to which participants used several tactics to find job openings (e.g., “used the internet to locate job openings”). Responses ranged from 1 (“very slightly or not at all”) to 5 (“very frequently”). |
| Faberman and Kudlyak [31] | Proprietary web-tracking data | Number of applications during each job search spell, defined as groups of searches separated by more than five weeks of inactivity. |
| Ochoa and Revilla [21] | Metered data | Time spent per day on job search platforms. |

In addition, we have included a paragraph highlighting the typical validation strategies applied in these studies.

“Beyond defining and measuring job search intensity, past research has also employed several validation strategies to evaluate the reliability and validity of the measures. Internal consistency reliability has been frequently assessed using Cronbach’s alpha or equivalent indices, with values above 0.70 indicating that items coherently capture the underlying construct [25, 27, 28, 29]. Factor analytic techniques, such as confirmatory factor analysis (CFA), have been used to examine whether items reflect a single latent construct [28]. These techniques —widely used in social sciences, psychology, and other disciplines— require proposing and testing multiple items, ultimately selecting a subset that forms a composite score intended to measure the concept of interest. Consequently, assessing the reliability and validity of single items is limited, as these methods primarily evaluate the quality of the construct as a whole. Moreover, extending such approaches to metered data raises challenges, since the equivalents of survey items are behavioral metrics, each recorded on distinct and often incomparable scales (e.g., visits, durations, or counts).

Predictive and concurrent validity have also occasionally been assessed, with studies showing that higher job search intensity scores are associated with higher success in finding a job [25, 27]. However, concurrent validity is based on associations measured at the same time, making it difficult to disentangle true relationships from shared-method variance. Predictive validity, in turn, is influenced by external factors that shape outcomes, meaning the evidence remains correlational and context-dependent. Furthermore, establishing similar forms of validity using metered data may be difficult when the predicted outcomes—such as employment success—cannot be directly observed within the digital traces themselves. This limitation could be addressed by complementing metered data with survey information collected from the same participants.

Together, these strategies illustrate the standard psychometric approach for establishing the soundness of survey-based measures of concepts such as job search intensity, as well as the methodological difficulties of adapting these approaches to metered data.”

Research Question and Contribution
Strengths:
• The research question is clearly stated and addresses a significant methodological issue.
• The contribution to the literature on digital trace data and multiverse analysis is well-positioned.
Suggestions for Improvement:
• Consider elaborating on the practical relevance of the findings for researchers in applied fields (e.g., labor economics, public policy).
• The contribution could be more explicitly framed in terms of advancing best practices for digital behavioral measurement.

Following your recommendation, we have revised the final part of this section to more clearly articulate the expected contribution of the study. The revised text emphasizes that this research aims to help scholars better understand the impact of different operationalization choices on the validity of findings derived from metered data, and to encourage further research on best practices for operationalizing concepts using such data:

“Beyond its methodological contribution, this research aims to advance best practices in digital behavioral measurement by illustrating how specific operationalization choices can influence substantive conclusions. It should help researchers using metered data and other forms of digital trace data better understand the limits and robustness of their results. Moreover, by identifying the main sources of variability and uncertainty, this study seeks to motivate future research to determine which operationalization factors are most critical for ensuring valid and comparable measures across studies.

The implications of this work extend beyond the study of job search behavior. As digital trace data are increasingly used across diverse disciplines—such as labor economics, public policy, communication studies, marketing, education, and computational social science—the insights provided here can help scholars in these fields develop more transparent, replicable, and theoretically grounded approaches to operationalizing behavioral constructs in digital environments.”

Data and Methods
Strengths:
• The data source (Netquest panel) and selection criteria are clearly described.
• The methodological rigor is commendable, with a systematic exploration of 10,080 operationalizations.
Suggestions for Improvement:
• Some operationalization decisions (e.g., thresholds for session separation or minimum time) appear arbitrary. Providing stronger theoretical or empirical justification would enhance credibility.
• The section is dense and technical; a summary table of operationalization factors and their levels is helpful but could be complemented with a flowchart or decision tree to aid comprehension.

We have added a justification of the operationalization factor levels. Wherever possible, we based the levels on prior literature or common practices, while also introducing plausible variation to assess sensitivity:

“The levels of operationalization factors were chosen to reflect prior literature or common practices where possible, while also exploring plausible variation. For instance, search spell separation time was defined around the five-week threshold used by Faberman and Kudlyak [31], with values of 0, 30, 60, and 90 days to test sensitivity. Session time follows the 30-minute standard used in Google Analytics that is also commonly applied in studies using metered data (e.g., [46]), with 10- and 60-minute alternatives used to capture shorter or longer sessions. Minimum time thresholds (2, 5, and 10 seconds) were inspired by Bosch [39], adapting page-level exposure thresholds to the job search context, where brief visits may suffice to evaluate a job posting. For other factors, plausible values were selected based on expected user behavior.”

To improve readability, we have added a flowchart (now Figure 1) that visually summarizes the process through which the different operationalizations of job search intensity are constructed. The figure illustrates how the eight metrics are obtained by combining two measurement types (time and visits) with four measurement targets (platforms, platform pages, job offer pages, and application pages), and how these are further diversified by applying multiple operationalization factors (e.g., platform type, session definition, minimum/maximum time thresholds, and outlier treatments). This visual aid complements Table 3 and helps readers more intuitively understand the combinatorial process that results in the large number of operationalizations examined in the study.

**Figure 1**

*Process of Constructing Multiple Operationalizations of Job Search Intensity Using Metered Data*

Results
Strengths:
• The results are presented in a structured and detailed manner, with appropriate use of statistical analyses and visualizations.
• The analysis of convergent validity and variability across metrics is thorough.
Suggestions for Improvement:
• The interpretation of statistical findings could be made more accessible to a broader audience. For example, what does a correlation of 0.14 vs. 0.91 imply in practical terms?
• Highlighting which metrics are most robust and why would be valuable for guiding future research.

Although the translation of different correlation levels to practical terms is not straightforward (i.e., multiple sets of values can produce the same correlation levels while leading to different substantive results), we have edited section “5.2 Variation Between Metrics” to better illustrate what can be expected from radically different correlation levels:

“The results show that while some metrics produce high average correlations between their measurements (e.g., M2-M6: 0.91), others are almost uncorrelated (e.g., M5-M8: 0.14). A correlation of 0.14 indicates virtually no linear relationship, meaning high values on one metric do not predict high values on the other; consequently, using one metric or the other could lead to opposite conclusions in practice. In contrast, a correlation of 0.91 suggests that the metrics capture very similar patterns of job search activity.”

We have also added a final paragraph in section “5.3 Variation Within Metrics” to state more clearly which metrics seem to be more robust:

“Overall, metrics M2 and M3 (time spent on pages and job offer pages) show the highest average correlations and relatively low variation across operationalizations, suggesting that they are more robust indicators of job search intensity. In contrast, metrics M4, M7, and M8 (time on applications, job offers, and applications counts) show higher variability, indicating greater sensitivity to operationalization choices.”

Discussion
Strengths:
• The discussion synthesizes the findings effectively and reflects on their methodological implications.
• The recognition of variability as a challenge in digital trace research is important and timely.
Suggestions for Improvement:
• The discussion could benefit from more concrete examples of how these findings might affect substantive conclusions in applied studies (e.g., gender differences in job search behavior).
• Consider offering more explicit recommendations for researchers on how to select and justify operationalizations.

We have edited the last paragraph of the section “6.1 Summary of Main Results” to provide more concrete examples of how substantive conclusions can be affected in applied studies:

“Importantly, these variations have practical implications for conclusions in substantive studies. For example, comparisons by gender show that most metrics suggest lower job search intensity among females, but the proportion of operationalizations producing statistically significant differences varies widely: 16.7% for M1 versus 0% for M4 and M6. Similarly, for age groups, older individuals generally exhibit lower search intensity, yet significance varies by metric (18.8% for M7 versus 0% for M2, M3, and M6). This illustrates that both the choice of metric and operationalization factors can influence not only the magnitude but also the statistical significance of observed differences, underscoring the need for careful metric selection in applied research.”

In addition, to provide more explicit guidance for applied research, we have edited section “6.3 Practical Implications” to include recommendations for metric selection and interpretation (see our response to your last comment below).

Limitations
Strengths:
• The limitations are acknowledged transparently, including the reliance on a single panel and metering technology.
Suggestions for Improvement:
• The implications of these limitations for generalizability could be discussed in more depth.
• Suggesting specific avenues for future research (e.g., cross-country comparisons, integration with survey data) would strengthen this section.

We have edited this section to state more explicitly the implications for the generalization of results:

“Consequently, the observed variability and robustness of job search intensity metrics may not generalize to other countries, labor markets, or cultural contexts, where online job search behavior and platform usage may differ. Moreover, studies addressing other research topics using metered data may exhibit different degrees of variability.”

We have also expanded the discussion of the main limitation of this study and outlined a potential avenue for future research to address it:

“Additionally, while this study examines how different operationalizations produce varying measurements and assesses their correlations as evidence of convergent validity, it does not evaluate other forms of validity, such as predictive validity, which could help assess which operationalizations better capture the concept of interest. Predictive validity could be examined by complementing metered data with survey data from the same individuals—an approach already employed by Bosch [30]. This is feasible when using metered panels built on regular survey panels, where participants provide both data types.”

Finally, as suggested, we have included a paragraph indicating potential future research directions to address these limitations:

“Future research could also replicate this analysis in other countries or labor markets, explore different metering technologies, and investigate how operationalization choices affect relationships with external variables such as employment outcomes, job offers, or job seeker well-being. Moreover, the same approach could be applied to other research topics to assess whether similar conclusions about metric variability and sensitivity to operationalization decisions hold.”

Practical Implications
Strengths:
• The manuscript recognizes that operationalization decisions can influence substantive findings.
Suggestions for Improvement:
• This section is relatively brief and could be expanded. What should researchers do when faced with multiple plausible operationalizations?
• Consider including a set of “best practice” guidelines or a checklist for operationalizing behavioral constructs using metered data.

We have extended this section with a set of considerations for operationalizing concepts using metered data, aimed at helping researchers make more informed decisions:

“More research is needed to establish a set of best practices for applied research when multiple plausible operationalizations are available. Such practices could include the following:

-	Ground operationalizations in theory and domain knowledge: Using knowledge about the topic can improve metric selection. Operationalizations should be theoretically justified and reflect meaningful effort. For example, time spent reading detailed job offers may better capture active job search than total pages viewed, while viewing job offers in general may reflect general interest in the labor market rather than genuine job search. Submitting applications may indicate actual job search effort.

-	Consider data availability: Even if some events better capture the concept of interest than others (e.g., applications better capture effort than viewing job offers), their observability among sampled individuals may limit their usefulness. For instance, the meter used in this study could not track within-app pages, restricting certain operationalizations.

-	Anticipate potential biases: Evaluate how metrics may over- or under-estimate behavior. For example, counting job offers viewed may overstate search activity if users browse casually, while counting sessions may inflate effort for users who log in frequently but engage superficially.

-	Account for operationalization factors and data corrections: Adjust thresholds and handle outliers according to the data and research question. Unusually long page durations may reflect inactivity rather than effort, making outlier correction more relevant for time-based metrics. Similarly, brief visits made only to check the labor market can be filtered by setting appropriate minimum durations per visit.

-	Validate with external data when possible: Comparing metered measures with survey data or other independent sources can help assess predictive or external validity and identify which metrics most accurately reflect the concept of interest.

-	Assess robustness: Computing multiple operationalizations, even using a limited set of options, can help evaluate the stability of results. As illustrated in this study, conclusions about gender or age differences in job search intensity may vary depending on the metric and operationalization used.”

Reviewer #2: Overall Assessment

The research question is interesting and timely. However, the execution of the study does not deliver sufficient analytical depth or theoretical contribution to warrant publication. The paper remains largely descriptive and does not demonstrate the broader implications or insights that could be gained from the analysis.

Major Comments

1. The results section primarily presents correlations and variability across operationalizations, but these are descriptive statistics without deeper interpretation. There is no clear advancement in theory, methodology, or substantive knowledge about job search behavior. Readers are left wondering: what do we actually learn about job search intensity beyond the fact that different metrics yield different numbers?

Thank you for your feedback. The main purpose of this paper is to raise awareness of potential issues that may arise when using digital trace data for research without a well-established method. Specifically, we aim to highlight: (1) the sources of error inherent in such data, and (2) the need for a formal process to operationalize concepts, analogous to established procedures in survey research (e.g., defining the concept, identifying dimensions, and selecting appropriate measures).

The rapid adoption of digital trace data has sometimes led researchers to treat it as a source of truth (e.g., Kobayashi & Boase, 2012; Junco, 2013; Araujo et al., 2017). We argue that this approach is misguided, and that descriptive research is necessary to estimate the magnitude of potential problems and assess the consequences of the current lack of methodological guidance. Our study provides empirical evidence on the variability and sensitivity of different operationalizations, illustrating the practical implications of these issues.

We fully acknowledge that the paper does not provide definitive solutions. Rather, our goal is to offer concrete evidence on the consequences of lacking well-established best practices and to motivate the development of such practices.

However, we have made several revisions to strengthen the paper’s practical value (see also answers to the next comments).

In particular, we now provide more information about how to interpret the different correlations presented in section “5.2 Variation Between Metrics”:

“The results show that while some metrics produce high average correlations between their measurements (e.g., M2-M6: 0.91), others are almost uncorrelated (e.g., M5-M8: 0.14). A correlation of 0.14 indicates virtually no linear relationship, meaning high values on one metric do not predict high values on the other; consequently, using one metric or the other could lead to opposite conclusions in practice. In contrast, a correlation of 0.91 suggests that the metrics capture very similar patterns of job search activity.”

Additionally, we now provide further interpretation on the variability/robustness in section “5.3 Variation Within Metrics”:

“Overall, metrics M2 and M3 (time spent on pages and job offer pages) show the highest average correlations and relatively low variation across operationalizations, suggesting that they are more robust indicators of job search intensity. In contrast, metrics M4, M7, and M8 (time on applications, job offers, and applications counts) show higher variability, indicating greater sensitivity to operationalization choices.”

References:

Araujo, T., Wonneberger, A., Neijens, P., & De Vreese, C. (2017). How Much Time Do You Spend Online? Understanding and Improving the Accuracy of Self-Reported Measures of Internet Use. Communication Methods and Measures, 11(3), 173–190. https://doi.org/10.1080/19312458.2017.1317337

Junco, R. (2013). Comparing actual and self-reported measures of Facebook use. Computers in Human Behavior, 29(3), 626–631. https://doi.org/10.1016/j.chb.2012.11.007

Kobayashi, T., & Boase, J. (2012). No Such Effect? The Implications of Measurement Error in Self-Report Measures of Mobile Communication Use. Communication Methods and Measures, 6(2), 126–143. https://doi.org/10.1080/19312458.2012.679243

2. While the paper shows variability, it does not translate this into meaningful implications for social science research or labor economics. For example, how should researchers decide among competing operationalizations? What guidance does this study provide for future measurement strategies? The discussion stops short of offering actionable recommendations.

We have made multiple improvements through the entire paper to make the implications of our results clearer.

In particular, we have edited the last paragraph of the section “6.1 Summary of Main Results” to provide more concrete examples of the implications of the variations for substantive conclusions:

“Importantly, these variations have practical implications for conclusions in substantive studies. For example, comparisons by gender show that most metrics suggest lower job search intensity among females, but the proportion of operationalizations producing statistically significant differences varies widely: 16.7% for M1 versus 0% for M4 and M6. Similarly, for age groups, older individuals generally exhibit lower search intensity, yet significance varies by metric (18.8% for M7 versus 0% for M2, M3, and M6). This illustrates that both the choice of metric and operationalization factors can influence not only the magnitude but also the statistical significance of observed differences, underscoring the need for careful metric selection in applied research.”

Moreover, in section “6.3 Practical Implications,” we now include a set of specific recommendations for researchers when operationalizing concepts using metered data:

“More research is needed to establish a set of best practices for applied research when multiple plausible operationalizations are available. Such practices could include the following:

-	Ground operationalizations in theory and domain knowledge: Using knowledge about the topic can improve metric selection. Operationalizations should be theoretically justified and reflect meaningful effort. For example, time spent reading detailed job offers may better capture active job search than total pages viewed, while viewing job offers in general may reflect general interest in the labor market rather than genuine job search. Submitting applications may indicate actual job search effort.

-	Consider data availability: Even if some events better capture the concept of interest than others (e.g., applications better capture effort than viewing job offers), their observability among sampled individuals may limit their usefulness. For instance, the meter used in this study could not track within-app pages, restricting certain operationalizations.

-	Anticipate potential biases: Evaluate how metrics may over- or under-estimate behavior. For example, counting job offers viewed may overstate search activity if users browse casually, while counting sessions may inflate effort for users who log in frequently but engage superficially.

-	Account for operationalization factors and data corrections: Adjust thresholds and handle outliers according to the data and research question. Unusually long page durations may reflect inactivity rather than effort, making outlier correction more relevant for time-based metrics. Similarly, brief visits made only to check the labor market can be filtered by setting appropriate minimum durations per visit.

-	Validate with external data when possible: Comparing metered measures with survey data or other independent sources can help assess predictive or external validity and identify which metrics most accurately reflect the concept of interest.

-	Assess robustness: Computing multiple operationalizations, even using a limited set of options, can help evaluate the stability of results. As illustrated in this study, conclusions about gender or age differences in job search intensity may vary depending on the metric and operationalization used.”

3. Much of the paper replicates or extends Bosch (2020) and Bosch & Revilla (2019) in another context. The novelty is limited to applying the same framework to job search data. Without a stronger theoretical rationale or a substantive insight about labor market dynamics, the contribution feels incremental.

To our understanding, given the very limited existing research on this topic (i.e., operationalization using metered data), extending the few available studies to new fields is essential before developing new methods to address this issue. Furthermore, we have expanded on previous work by exploring additional factors that may affect operationalization variability (e.g., treatment of outliers, segmentation of online activity into spells). In addition, following our earlier responses, we have added a section with practical recommendations, which we expect will add further value for readers.

In Section “3. Research Questions and Contribution,” we have clarified the main purpose and potential contribution of the paper as follows:

“Beyond its methodological contribution, this research aims to advance best practices in digital behavioral measurement by illustrating how specific operationalization choices can influence substantive conclusions. It should help researchers using metered data and other forms of digital trace data better understand the limits and robustness of their results. Moreover, by identifying the main sources of variability and uncertainty, this study seeks to motivate future research to determine which operationalization factors are most critical for ensuring valid and comparable measures across studies.

The implications of this work extend beyond the study of job search behavior. As digital trace data are increasingly used across diverse disciplines—such as labor economics, public policy, communication studies, marketing, education, and computational social science—the insights provided here can help scholars in these fields develop more transparent, replicable, and theoretically grounded approaches to operationalizing behavioral constructs in digital environments.”

4. The paper repeatedly acknowledges that it cannot assess which operationalization is “valid.” Yet without any form of external validation (e.g., predictive validity against employment outcomes or survey responses), the exercise becomes self-contained and somewhat circular. This limits the value of the findings.

Although further validity analyses are not feasible with the available data, we have revised the manuscript to provide more information about validation strategies. First, we added a paragraph in section 2.1 “Operationalization of Job Search Intensity” that summarizes the strategies used in previous research and their limitations.

“Beyond defining and measuring job search intensity, past research has also employed several validation strategies to evaluate the reliability and validity of the measures. Internal consistency reliability has been frequently assessed using Cronbach’s alpha or equivalent indices, with values above 0.70 indicating that items coherently capture the underlying construct [25, 27, 28, 29]. Factor analytic techniques, such as confirmatory factor analysis (CFA), have been used to examine whether items reflect a single latent construct [28]. These techniques —widely used in social sciences, psychology, and other disciplines— require proposing and testing multiple items, ultimately selecting a subset that forms a composite score intended to measure the concept of interest. Consequently, assessing the reliability and validity of single items is limited, as these methods primarily evaluate the quality of the construct as a whole. Moreover, extending such approaches to metered data raises challenges, since the equivalents of survey items are behavioral metrics, each recorded on distinct and often incomparable scales (e.g., visits, durations, or counts).

Predictive and concurrent validity have also occasionally been assessed, with studies showing that higher job search intensity scores are associated with higher success in finding a job [25, 27]. However, concurrent validity is based on associations measured at the same time, making it difficult to disentangle true relationships from shared-method variance. Predictive validity, in turn, is influenced by external factors that shape outcomes, meaning the evidence remains correlational and context-dependent. Furthermore, establishing similar forms of validity using metered data may be difficult when the predicted outcomes—such as employment success—cannot be directly observed within the digital traces themselves. This limitation could be addressed by complementing metered data with survey information collected from the same participants.

Together, these strategies illustrate the standard psychometric approach for establishing the soundness of survey-based measures of concepts such as job search intensity, as well as the methodological difficulties of adapting these approaches to metered data.”

Second, we have also expanded the discussion of the main limitation of this study and outlined a potential avenue for future research to address it:

“Additionally, while this study examines how different operationalizations produce varying measurements and assesses their correlations as evidence of convergent validity, it does not evaluate other forms of validity, such as predictive validity, which could help assess which operationalizations better capture the concept of interest. Predictive validity could be examined by complementing metered data with survey data from the same individuals—an approach already employed by Bosch [30]. This is feasible when using metered panels built on regular survey panels, where participants provide both data types.”

Despite these acknowledged limitations, we remain confident in the value of the research and expect that the changes and additions made following this review will further strengthen its contribution.

Reviewer #3: Introduction

• “The research problem is stated clearly, but it is not well justified that research is needed.” Please elaborate more and provide some arguments and support with references.
• “Language problems make it difficult to follow at times.”
• In Part 2.2, please support with more references.

Thank you for your feedback. Research using digital trace data, and particularly metered data, is still relatively new. Consequently, methodological studies in this area remain scarce compared with those based on more established data sources such as surveys. This scarcity of methodological work constitutes the main motivation for our paper. The adoption of metered data as an innovative source has often been accompanied by the assumption that it provides superior data quality compared to surveys, sometimes being treated as a “source of truth” for studying online behaviors (e.g., [8 – 10]). Our research seeks to challenge this assumption by highlighting its limitations and emphasizing the need for methodological frameworks equivalent to those long established for survey data.

This gap is reflected in section 2.2, where we could not identify additional studies examining job search behavior using metered data or other forms of digital trace data. However, we have extended this section with literature about research using metered data to give a broader context to readers:

“Digital trace data are increasingly used to study a wide range of online behaviors and their societal implications. For instance, they have been employed to predict voter turnout based on online activity [33], examine Facebook’s impact on the public agenda [34], analyze echo chambers in online news consumption [35], investigate how exposure to “fake news” shapes political attitudes [36], and explore the relationship between online vaccine-related information consumption, social media use, and attitudes toward vaccines [37]. These examples illustrate the growing methodological relevance of digital trace data across social and political sciences.”

References:

8.	Kobayashi, T., & Boase, J. (2012). No Such Effect? The Implications of Measurement Error in Self-Report Measures of Mobile Communication Use. Communication Methods and Measures, 6(2), 126–143. https://doi.org/10.1080/19312458.2012.679243

9.	Araujo, T., Wonneberger, A., Neijens, P., & De Vreese, C. (2017). How Much Time Do You Spend Online? Understanding and Improving the Accuracy of Self-Reported Measures of Internet Use. Communication Methods and Measures, 11(3), 173–190. https://doi.org/10.1080/19312458.2017.1317337

10.	Junco, R. (2013). Comparing actual and self-reported measures of Facebook use. Computers in Human Behavior, 29(3), 626–631. https://doi.org/10.1016/j.chb.2012.11.007

33.	Bach, R.L., Kern, C., Amaya, A., Keusch, F., Kreuter, F., Hecht, J., Heinemann, J., 2019. Predicting Voting Behavior Using Digital Trace Data. Social Science Computer Review 089443931988289. https://doi.org/10.1177/0894439319882896

34.	Cardenal, A.S., Galais, C., Majó-Vázquez, S. (2018). Is Facebook Eroding the Public Agenda? Evidence from Survey and Web-Tracking Data. International Journal of Public Opinion Research. https://doi.org/10.1093/ijpor/edy025

35.	Cardenal, A. S., Aguilar-Paredes, C., Cristancho, C., & Majó-Vázquez, S. (2019). Echochambers in online news consumption: Evidence from survey and navigation data in Spain. European Journal of Communication, 34(4), 360-376. https://doi.org/10.1177/0267323119844409

36.	Guess, A. M., Lockett, D., Lyons, B., Montgomery, J. M., Nyhan, B., & Reifler, J. (2020). "Fake news" may have limited effects beyond increasing beliefs in false claims. Harvard Kennedy School Misinformation Review, 1(1). https://doi.org/10.37016/mr-2020-004

37.	Guess, A. M., Nyhan, B., O'Keeffe, Z., & Reifler, J. (2020b). The sources and correlates of exposure to vaccine-related (mis)information online. Vaccine, 38(49), 7799– 7805. https://doi.org/10.1016/j.vaccine.2020.10.018

Regarding the reported language problems, we have carefully reviewed and edited the entire manuscript with the support of a native English speaker to improve clarity and readability across all sections.

Literature Review

• The current research lacks a more elaborate literature review section and supported references.
• Please elaborate more on the literature review about the operationalization factors of “job search intensity” based on current and previous research, and add the arguments to support your research if applicable.

In section “2.1 Operationalization of Job Search Intensity,” we have added a new reference (Blau, 1993) and summarized the various operationalizations of job search intensity found in the literature, including the factors used in these operationalizations, in a new Table 1:

**Table 1**

*Different operationalizations of job search intensity in the literature*

| **Authors** | **Data used** | **Operationalization** |
| --- | --- | --- |
| Ellis et al. [24] | Paper survey | Multidimensional construct including time and effort spent, and number of employers contacted. |
| Blau et al. [25] | Paper survey (postal) | Four items assessing general effort (e.g., “Devoted much effort to looking for a job.”), rated on a 5-point Likert scale (“Strongly Disagree” to “Strongly Agree”). |
| Barber et al. [26] | Paper survey (in person and postal) | Two different operationalizations were used:   3. Total number of job search sources used (0 to 8). 4. An operationalization based on two questions:  - Time spent, measured either (1) through an open question asking for hours per week, or (2) via a scale ranging from “one hour or less” to “more than twenty hours.” - Subjective effort, rated on a 5-point scale from “very little effort” to “a great deal of effort.” |
| Saks and Ashforth [27] | Paper survey (postal) | Four items assessing general effort (e.g., “Spent a lot of time looking for job opportunities”), rated on a 5-point Likert scale (“Strongly Disagree” to “Strongly Agree”). |
| Wanberg et al. [28]  Zacher [29] | Paper survey (in person and postal) | Seven items, including "used the Worldwide Web or other computer services to locate job openings". Response options ranged from 1 (“never or zero times”) to 5 (“very often, at least ten times”). |
| da Motta Veiga [30] | Online survey | Four items assessing the extent to which participants used several tactics to find job openings (e.g., “used the internet to locate job openings”). Responses ranged from 1 (“very slightly or not at all”) to 5 (“very frequently”). |
| Faberman and Kudlyak [31] | Proprietary web-tracking data | Number of applications during each job search spell, defined as groups of searches separated by more than five weeks of inactivity. |
| Ochoa and Revilla [21] | Metered data | Time spent per day on job search platforms. |

As mentioned in the previous response, in section “2.2 Operationalizing Concepts Using Digital Traces,” five new references have been added to provide context on common applications of metered data.

Research Methodology
• In the Research Methodology section, the authors should identify the research type (qualitative or quantitative), the sampling procedures, and why they used each sampling method. Additionally, this section should briefly explain and reference the analytical tools.

All analyses described in section “4.3 Analyses” are strictly quantitative. The sampling procedure and its rationale—specifically, the selection of panelists from a metered panel using predefined criteria to ensure good coverage of metered data across the entire period of interest—are detailed in section “4.1 Data.”:

“The final dataset comprises 600 panelists, selected from a pool of 18,980 metered panelists in Spain. The selection criteria included providing metered data throughout the entire study period, having visited at least one of the job search platforms of interest on at least five different days, and having previously supplied demographic information on gender, age, region, and social class. From this eligible group, 600 panelists were randomly selected.”

The analytical tools used are described in section “4.3 Analyses.” These include assessing correlations between pairs of measures grouped according to different criteria to address the research questions, analyzing the distribution of pairwise correlations, computing the mean and standard deviation of correlations sharing the same basic metrics, and using mixed-effects linear regressions to evaluate the impact of various factors on overall variation.

Analysis and Discussion
• You need to state the significance of the R model analysis used in the research.
• Findings just report the results; there is no critical discussion. The analysis needs to be more deeply backed by research evidence and focus on the impact of the results.

First, we have expanded the regression analyses in section “5.4 Effect of Operationalization Factors on Measurements for Each Metric” by including three additional metrics to assess model fit: AIC, BIC, and RMSE. The significance of each regression coefficient was already indicated with an asterisk (*) at the 5% significance level.

Regarding the presentation of results, the section has been revised to help readers better interpret the meaning and relevance of the numerical findings. For example, rather than only reporting correlation values, we now clarify their implications, as follows:

“The results show that while some metrics produce high average correlations between their measurements (e.g., M2-M6: 0.91), others are almost uncorrelated (e.g., M5-M8: 0.14). A correlation of 0.14 indicates virtually no linear relationship, meaning high values on one metric do not predict high values on the other; consequently, using one metric or the other could lead to opposite conclusions in practice. In contrast, a correlation of 0.91 suggests that the metrics capture very similar patterns of job search activity.”

Finally, we have added a paragraph discussing the results and their implications at the end of this section:

“Overall, metrics M2 and M3 (time spent on pages and job offer pages) show the highest average correlations and relatively low variation across operationalizations, suggesting that they are more robust indicators of job search intensity. In contrast, metrics M4, M7, and M8 (time on applications, job offers, and applications counts) show higher variability, indicating greater sensitivity to operationalization choices.”

Conclusion
• The discussion section should be elaborated on more and supported by related references (it was too short, and there was no synthesis explanation).
• Implications are included, but future directions are underdeveloped in this section.

This section has been substantially expanded to include recommendations for researchers seeking to operationalize concepts of interest using metered data:

“More research is needed to establish a set of best practices for applied research when multiple plausible operationalizations are available. Such practices could include the following:

-	Ground operationalizations in theory and domain knowledge: Using knowledge about the topic can improve metric selection. Operationalizations should be theoretically justified and reflect meaningful effort. For example, time spent reading detailed job offers may better capture active job search than total pages viewed, while viewing job offers in general may reflect general interest in the labor market rather than genuine job search. Submitting applications may indicate actual job search effort.

-	Consider data availability: Even if some events better capture the concept of interest than others (e.g., applications better capture effort than viewing job offers), their observability among sampled individuals may limit their usefulness. For instance, the meter used in this study could not track within-app pages, restricting certain operationalizations.

-	Anticipate potential biases: Evaluate how metrics may over- or under-estimate behavior. For example, counting job offers viewed may overstate search activity if users browse casually, while counting sessions may inflate effort for users who log in frequently but engage superficially.

-	Account for operationalization factors and data corrections: Adjust thresholds and handle outliers according to the data and research question. Unusually long page durations may reflect inactivity rather than effort, making outlier correction more relevant for time-based metrics. Similarly, brief visits made only to check the labor market can be filtered by setting appropriate minimum durations per visit.

-	Validate with external data when possible: Comparing metered measures with survey data or other independent sources can help assess predictive or external validity and identify which metrics most accurately reflect the concept of interest.

-	Assess robustness: Computing multiple operationalizations, even using a limited set of options, can help evaluate the stability of results. As illustrated in this study, conclusions about gender or age differences in job search intensity may vary depending on the metric and operationalization used.”

Additionally, section “6.2 Limitations” has been extended to provide a more detailed discussion of directions for future research:

“Future research could also replicate this analysis in other countries or labor markets, explore different metering technologies, and investigate how operationalization choices affect relationships with external variables such as employment outcomes, job offers, or job seeker well-being. Moreover, the same approach could be applied to other research topics to assess whether similar conclusions about metric variability and sensitivity to operationalization decisions hold.”
